# Narratives of recovery from persistent fatigue: a stepwise learning process

**DOI:** 10.1080/02813432.2026.2637743

**Published:** 2026-02-28

**Authors:** Hannah Linnros, Anna Andreasson, Anna-Karin Norlin, Lars-Christer Hydén

**Affiliations:** a Clinical Department of Pain and Rehabilitation, Region Östergötland, Linköping, Sweden; b Department of Health, Medicine and Caring Sciences, Linköping University, Linköping, Sweden; c Department of Psychology, Stockholm University, Stockholm, Sweden; d Department of Clinical Neuroscience, Karolinska Institutet, Stockholm, Sweden; e Department of Culture & Society, Linköping University, Norrköping, Sweden

**Keywords:** Cfs/ME, exhaustion disorder, Post COVID-19 condition, transdiagnostic, personal experience

## Abstract

**Background:** Persistent fatigue is a transdiagnostic symptom that is present in many different medical conditions and diagnoses and is a common reason for seeking health care. Despite a lack of consensus on how to understand and treat persistent fatigue, a subset of patients recover. The experiences of patients who recovered from persistent fatigue have important implications for future research and rehabilitation interventions. **Purpose:** This study aimed to further improve the understanding of the recovery process for people who have improved function and regained health following three health conditions characterized by persistent fatigue (CFS/ME, Post Covid-19 Condition and Exhaustion Disorder), and to develop a comprehensive model of the recovery process. **Methods:** Fourteen former patients shared their stories about their recovery during videotaped interviews. Narrative analysis was used to explore participants’ experiences of the recovery process, focusing on decisive events. **Results:** Recovering from persistent fatigue could be understood as a nonlinear stepwise learning process with a marked turning point. This turning point involved finding hope and a new understanding of their fatigue condition, which in turn provided the patient with guidance on what measures to take. **Discussion:** We discuss the results in relation to previous research as well as their clinical implications. A new understanding of the symptoms seems to be central to recovery from persistent fatigue, but it is not sufficient in itself. Health care needs to provide explanatory models that fuel hope and agency, as well as individualized interventions, to enable the recovery process.

## Introduction

The term fatigue is used in medical settings to describe tiredness that is not alleviated by rest [1]. Persistent fatigue is present in many different medical conditions and is a common reason for seeking health care [1–4]. Despite being a common and debilitating symptom [5], and a number of studies showing improvement in fatigue patients after cognitive behavioral therapy [6–8], there is a lack of consensus regarding the etiology and treatment of persistent fatigue [9]. Increasing evidence that the same cognitive-behavioral responses to fatigue moderate and mediate treatment outcomes regarding fatigue across conditions [8] implicates that the recovery process from fatigue might share common transdiagnostic features. This points to the opportunity to gain meaningful knowledge from persons who have managed to recover from different fatigue conditions, and whether there might be common patterns in their recovery processes that could inform clinical practice.

To date, very little research has been conducted on recovery from persistent fatigue. We have selected to look closer at the recovery process in people who had previously been diagnosed with three medical conditions characterized by persistent fatigue: Chronic Fatigue Syndrome (CFS)/Myalgic Encephalomyelitis (ME), Exhaustion Disorder (ED) and Post-COVID-19 condition (PCC). Both CFS/ME and PCC are considered post-infectious fatigue syndromes, and during and after the COVID-19 pandemic, many have reported persistent fatigue despite mild disease [10–12]. In ED, a locally used diagnosis part of the Swedish version of the International Classification of Disease ICD-10-SE, long-term stressors are considered central to symptom etiology [13]. All three conditions have in common that they are diagnosed based on anamnestic case definitions and there are as of yet no biomarkers or medical investigations that can confirm diagnosis. The lack of consensus on how to interpret existing evidence regarding etiology or interventions makes all three diagnoses more or less contested [9,14].

Since the 1990s, several studies have focused on patient experiences of persistent fatigue [15–17], and in recent years, fatigue as part of PCC [18] has further increased this interest. Some qualitative studies have focused specifically on illness narratives (see, for instance [14,19–21]). To study stories about illness experiences, illness narratives, is a way to let patients’ voices on experiences of suffering and vulnerability be heard, which in turn can lead to an improved understanding of how patients try to manage their changed life situation. Such narratives also highlight the reconstruction of identity that often accompany chronic diseases and persistent symptoms [22,23]. So far few studies have focused on the recovery processes in persistent fatigue. To improve the understanding of fatigue conditions and what might facilitate recovery, recovery narratives must be researched. Studying recovery narratives could increase the knowledge of what further research on treatment is needed and, in the long term, what rehabilitation interventions should be offered to patients. It may also shed light on the social and structural aspects of the recovery process, including the promoting and hindering factors.

### Recovery from persistent fatigue

Recovery can be conceptualized in multiple ways. In many clinical settings, recovery is understood in a disease-oriented manner, where it is seen as remission or cure as the result of a natural biological healing process or an effective treatment, and where health is defined as the absence of disease [24]. However, the majority of diseases in health care are chronic conditions, such as cardiovascular disease, cancer, diabetes and other metabolic disorders and dementia. As pointed out by Mengshoel, this view on recovery leaves little hope for patients, which may in turn hamper them from actively bringing wellness into their lives [25]. This more biomedical view on recovery is also found in some mental health research, where clinical recovery refers to the cure of all symptoms of mental diseases [26].

An alternative way to understand recovery is as a personal recovery process. From this perspective, recovery is conceptualized in terms of a personal experiential process focusing on illness experience and the process of overcoming or coming to terms with illness in real-life situations [25].

Since the 1990s, personal recovery has been one of the main focuses of research on mental health recovery. It is about the complex process of making life meaningful and includes healing, empowerment, and reconnection with the social world, as well as a process of personal growth and transformation of the self [25,27]. In mental health research a variety of narrative approaches have been used to study recovery as experienced, described, and presented. In a systematic review, Llewellyn-Beardsley et al. [28] showed that mental health recovery narratives are multidimensional, in a way that they express personal meaning-making processes at the same time as they present health-promoting and hindering factors at the individual, socio-cultural, and systemic levels. Such narratives are often nonlinear, describing a process with both turning-points and feedback loops [28].

To date, only a few studies have focused on personal recovery within fatigue recovery research, and there are no systematic reviews. Existing research has mainly focused on patients diagnosed with CFS/ME and presents a perception of recovery as not primarily about being free of symptoms but rather about getting rid of the sick role and regaining a sense of normality [29]. With time, new perspectives and coping strategies evolve in relation to fatigue, with a new balance between fatigue and everyday life [30]. Hasan et al. [31] found that due to the lack of health care support, patients independently constructed and managed their own treatment plans. Experiences of dismissive and stigmatized responses from clinicians precipitated disengagement from the medical system and a turn to other forms of treatment, often mind-body approaches. In a study on patients’ reflections a decade after receiving a diagnosis of ED, rehabilitation was described as the start of a personal development towards a more authentic self-image and a long-lasting process of re-evaluating learned behavior and thought patterns [32].

To the best of our knowledge, only three fatigue recovery studies have used a narrative approach, all on patients recovered from CFS/ME. Bakken et al. [33] identified distinct turning points in the illness narratives, implying a profound narrative shift and change in mindset, followed by long-term work to actively pursue their own healing. Their understanding of being helpless victims of the disease was replaced by a changed view of causality and illness and a new sense of self-agency. In another study, Krabbe et al. [34] emphasized the importance of finding ways to gain bodily knowledge and trust from new experiences, although they did not place as much emphasis on a transformative narrative shift. Brown et al. [35] found that the recovery process was described in terms of individual effort and responsibility, and that individuals were prepared to try anything that might be beneficial. A comprehensive model of the personal recovery process, grounded in patients’ narratives, remains a significant gap in the fatigue research literature.

### Aim and research questions

This research emerged from questions raised in a context of transdiagnostic, clinical work with patients experiencing persistent fatigue. The overall aim of the study was to deepen the understanding of the recovery process for patients who have improved function and regained health following health conditions characterized by persistent fatigue. A further objective was to develop a comprehensive model of the recovery process. The research questions were as follows:

- How do the participants describe the recovery process?
- Which events and factors do participants describe as decisive in the recovery process?

## Methods

Narrative approaches have previously been employed in studies of recovery processes from fatigue as well as in studies of mental health recovery (see for instance [19,28,33,34]). This study focused on recovery as a process and specifically examined how interviewees described their experiences of recovery. Since individuals often convey change over time through storytelling, a narrative approach was chosen both to (i) elicit storytelling in the interviews and (ii) to analyze these stories in such a way that it was possible to identify the central events in the stories and the relation between these events [36–38]. This form of narrative analysis draws on classical theories about oral storytelling [39] and analytical endeavors focusing on the organization of narrative events in interview elicited narratives with the aim to identify core event patterns [40,41]. Through this approach, it becomes possible to develop a comprehensive model describing the recovery process.

### Participant recruitment and sampling

Data were collected in Sweden from March 2023 to October 2024. The sampling was purposeful and criteria-based. All participants were adults and met the following inclusion criteria.

- Previously diagnosed with CFS/ME, ED, or PCC. The diagnosis was verified using medical record excerpts.
- Having been on sick leave for least 50% for at least six months with persistent fatigue as main symptom. Two participants were too young to be working when they developed fatigue and, therefore, had not been on sick leave. Since they had been bedridden for long periods, we concluded that their low level of functioning was equivalent to fulfilling this criteria.
- Having fully recovered from their persistent fatigue condition, defined as subjectively seeing themselves as recovered and objectively working or studying at least 75% of full time. For most patients, return-to-work is perceived as a central element of the personal recovery process [42].

We aimed to achieve equal group sizes for the three diagnoses and a gender distribution mirroring the overrepresentation of women in all three diagnostic groups [43–45], and we actively ensured that we would interview at least one man from each group. Two participants turned out to have double diagnoses. Participants were recruited through different formal and informal networks. Health care staff and other researchers were informed about the study and asked to help identify potential participants and ask them permission to be contacted for the study. Participants were also recruited from social media platforms for people who had recovered from severe fatigue. There was also snowball sampling in which participants suggested other participants. The sampling was conducted with the purpose of attaining as much variation as possible in the background of education and recovery methods and experiences. None of the participants had a clinical relationship with the authors.

Potential participants were contacted *via* e-mail or phone. They were provided with oral and written information about the study, and participants provided written and video-taped informed consent. No potential participants who were contacted dropped out, but two persons were excluded as the interviews revealed that they did not consider themselves recovered, thereby not fulfilling all inclusion criteria.

Interviews with 14 participants (nine women, four men and one other median age 36 years) were included in the study (see Table 1). The median duration of illness from diagnosis was 32 months and the median time since full recovery was 10 months at the time of the interview. It was often difficult for participants to date the symptom debut; in these cases, the illness duration was counted from the time of diagnosis, which means that the actual illness duration would be longer. Several participants had been bedridden or homebound for years. Two participants diagnosed with ED found interventions provided by health care helpful in their recovery process. However, most interviewees sought help outside the public health care system, searching the internet for information on how to get well. Several participants had successfully used online programs, while others had been trying different strategies on their own.

**Table 1.** Overview of all participants with social background and diagnosis.

*Partici-pant #*	*Age*	*Gender*	*Social situation at illness onset*	*Education*	*Diagnosis*	*Illness duration in years from diagnosis*
1	50s	Woman	Working, living with partner and small children	Secondary school + 1 yr	CFS/ME	3
2	30s	Other	Working. In relationship, living alone	University	CFS/ME	7
3	30s	Woman	Just graduated from university, working, living with partner	University	ED, CFS/ME	3,5
4	20s	Woman	University student, living alone	Not finished university studies	CFS/ME	2,5
5	30s	Man	Just graduated from secondary school, living with parents	Secondary school + folk high school	CFS/ME	11
6	30s	Woman	Working, living with partner	University	ED	1,5
7*	40s	Woman	Working, living with partner and small children	University	ED	6
8	50s	Man	Working, living with partner and school age children	University	ED	1
9*	30s	Woman	Working, living with partner	University	ED	1
10*	30s	Man	Working, living with partner	University	ED, PCC	4,5
11	50s	Woman	Working, living with partner and school age children	University	PCC	1
12*	40s	Woman	Working, living with partner and small children	University	PCC	1
13	30s	Woman	Working, living with partner and small children	University	PCC	1,5
14*	60s	Man	Working, divorced, living with teenage children and new partner	University (2 yrs)	PCC	3,5

* online interview.

### Narrative interviews

The first author conducted all the interviews. The goal was to conduct as many face-to-face interviews as possible, but due to long travel distances, five interviews were conducted *via* a digital video communication platform. For face-to-face interviews, the participants could choose to be interviewed either in their homes or at a place suggested by the interviewer. No non-participants were present during the interviews. A pilot interview was conducted and was included in the main study. All interviews were video-recorded to capture body language and other information that could not be obtained through audio recording only.

Before the interview, participants were informed that the focus of the interview would be their recovery process and the events, factors, and circumstances they considered important. They were also informed that the interviews would be 45–60 min long. Before or during the interview, the interviewees were asked to provide standard background information on a written form (gender, age, family situation, educational level, current occupation or studies, onset of symptoms, time and type of diagnosis, and month of recovery).

Each interview began with the interviewer introducing herself and repeating the aim of the study. The interviews followed a narrative approach [36], initiated by an open-ended question inviting the interviewee to describe how they had recovered from their fatigue condition. Interviewees were encouraged to tell their stories in their own preferred way, while the interviewer minimized interruptions. If key topics related to the research question had not been addressed spontaneously, further open-ended questions were posed to explore those areas. The interviews lasted for 16–62 min, with a median duration of 45 min. All were transcribed and anonymized by the first author. All participants were offered to read and comment on their transcribed interviews afterwards, but no comments were added after reading.

### Narrative analysis

In response to the interviewer’s question about how they had recovered from their fatigue condition, all interviewees started to tell a chronologically structured story about their recovery process. In all cases the stories were organized around sequences of events that unfolded as the interviewee sought to understand and cope with the fatigue.

The analysis of the interviews was based on identifying the central events in the stories told by the interviewees, the relationship between these events in each interview, and a systematic comparison of the event structure between the individual stories told by the interviewees [37,46]. This was done in six steps: (i) All interviews were *transcribed* verbatim with special notice of pauses and hesitations. (ii) All the authors *read* the transcribed interviews repeatedly to familiarize themselves with the data. (iii) In each interview, all *events* that were described as part of the recovery process were identified by first and last author. Events were identified through the interviewees’ use of verbs (including mental verbs) connected to changes (for instance, deciding to begin with physical activities, or engaging in online activities). (iv) *Codes* for the domains in which events took place were developed (for instance, “health care encounters”, “the private sphere”, “social media”, or “workplace related”) and events that marked a turning point in the process were identified. NVivo software (version 14.23.4) was used for coding the material. Coding was done jointly by first and last author. (v) For each interview, the events were *arranged* chronologically to obtain an overview of each participant’s recovery process. (vi) Patterns in the chronological chains of events were first identified in the individual interview and then *compared* across all interviews in search for shared patterns in the stories, as well as exceptions to such patterns.

## Ethics

In accordance with national legislation, the study was approved by the responsible ethical review board before it commenced (Dnr 2022-07329-01) and was conducted in accordance with the Helsinki Declaration Act. Written and video-taped informed consent was obtained from all the participants prior to each interview. All data were stored on a high-security server and could only be accessed by the authors. The data used in this study have been anonymized and presented in a way that minimizes the risk of identifying individual participants.

## Results

The analysis of the interview stories told by the participants revealed that the narratives were constructed and told around a sequence of events that, for most participants, extended across several years and taken together described the individual’s recovery process. The narratives were chronologically ordered from the “beginning” to the “end” to the time of the interview. For most participants, the story began with becoming ill or already being ill for a long time. The events constituting the recovery process had to do with either things that happened to the interview person or, more frequently, decisions they took and the actions they performed. Some events had the character of being described as “turning-points” in the recovery process. All events were generally well described and delineated by the interviewees.

An analysis of the events and their chronological order showed that the events could be grouped into a stepwise process, with several distinct *steps* following each other. The patterns in these steps were never described as being preordained, but rather as following a “pragmatic logic”: the interview person did what they considered to be possible in the given situation and continued to do what “worked.” What was considered possible was to a high degree redefined through the recovery process itself as when the interviewees opened new possibilities through their own “successful” actions.

In the analysis, five major chronologically ordered steps were identified: (1) the start and the health impasse; (2) quest; (3) turning point; (4) searching, testing, and evaluating; and (5) regaining a life. These steps are described below and illustrated with quotations from the interviews. Minor deviations from this structure are discussed in the text.

### The start and the health impasse: stuck in a hopeless situation

Some interviewees started their story with their illness debut, while others started after extended contact with health care. With the exception of three interviewees, all described the starting point of the recovery process as a situation characterized by them still being ill but running out of possible actions that could lead to health improvements. The participants described how their fatigue and other related symptoms had become severely debilitating, making it impossible to continue their ordinary work, take care of their households, and have the social life they wanted.

All participants had been in contact with health care, often a primary care center, and sometimes specialized clinics. Experiences of the health care system are generally described as negative despite several visits. The participants described having a range of medical investigations that resulted in little helpful advice. In the end, the participants were, in most cases, given a diagnosis, but not much more. Even though some doctors were described as empathetic and supportive, they seldom provided answers or hope. This resulted in suffering, despair, and hopelessness for almost all participants.

*She, the first doctor I met, the first thing she said to me was like: “well no, one does not recover from this”. And at that time I was 21. And that is, that is just like GIVING someone a depression. (#4*)

Those participants who had received a diagnosis of CFS/ME or PCC described how they were told that there was no cure or that knowledge about treatment options was unavailable. Media coverage of the pandemic and the remaining symptoms after COVID-19 contributed to the fear of never getting well in patients with PCC.

*The media images were rather (.) gloomy and then there was this image like: 80 per cent get a mild infection, 20 per cent do not get a mild infection, or get seriously ill. So that image kind of got stuck, that I am one of those that will get seriously ill. And I remember the same day that I got ill there was a coverage on, on TV, of a young person that just had become-, that couldn’t breathe. There was like a fright there in the beginning of the whole pandemic. But then it was that I nurtured this idea of “What if I’m never going to get well”. So that idea was there the whole time and it was a – I think it was what stopped the body from healing or what one should call it. I am not sure exactly what had happened in my body, but that’s what it felt like. (#12)*

For many participants, the lack of options for action led to frustration and a sense of hopelessness, not knowing if recovery was a realistic outcome. In this desperate situation, three interviewees expressed thoughts about either ending their lives or not caring about whether they lived or died.

*It gets very dramatic now, but I gave myself a final date. One year. I would work on this for one year. And if I managed to get better, well, well then that would be fine. But if I wouldn’t succeed, I didn’t want to go on living anymore. (#4)*

Not understanding what caused the symptoms or knowing what to expect from the next health care encounter and of their future life adds to further suffering and stress. For several participants, this eventually led to cessation of seeking health care. They had entered a health impasse. Two participants recovered from ED who described receiving helpful explanations of their fatigue condition from health care did not describe the same experience of hopelessness or health impasse.

*So, the doctor could go through what happens to the body during a challenge and confirmed that (1s) this yes, this is exhaustion disorder and uhm, explained that the symptoms really matched the ones you usually get. And that in itself was a relief, just to learn what was happening with the body. (#9)*

### Going on a quest for recovery

The impasse situation, characterized by a sense of hopelessness and despair, resulted in attempts to do something, to find possibilities, and not literally succumb to the illness. This led the interviewees to the next step in their recovery process, defined through several events having to do with seeking information about the illness and various possibilities for improving their health.

The participants had typically already spent much effort trying to get well, but their efforts did not lead to what they hoped for. For most of the interviewees, the suffering and feelings of hopelessness and helplessness are described as so unbearable that they are ready to do anything to change the situation. The participants who described their thoughts about ending their lives talked about making a choice to try to find alternatives.

*And then I had to see another doctor, I think it was at the same primary health care center, who also took about the same blood tests and said about the same things, which also was hard, since I still didn’t know what to do. [….] But it was difficult not getting any answers. What is happening in my body? What is HAPPENING to me? It was really hard, and it opens up for, that you want to find answers in other ways, somehow. (#12)*

When health care could not provide answers or effective treatment, participants described how they thought it was necessary to look elsewhere for opportunities to do something to change their situation; that is, to start a quest to find ways to improve their health through their own activities. Almost all interviewees described how they started seeking information on the internet.

*Well, so it was like just a kind of survival mode until I could start googling a bit. I couldn’t even watch a phone. It was like – I got so dizzy and vomited just by holding the phone and watch. But since I didn’t do anything else I could kind of watch for 20 s and then rest for a minute and then watch another 20 s. And then I could start googling a bit on this. Because at that time I had gotten autonomic dysfunction, all doctors agreed on that. Even if no one said how I could cure it, or how I could get well. (#13)*

The participants described how they found both private health care providers and social communities on the internet, offering new directions and opportunities for agency so they could themselves do something to improve their health. When the quest for recovery began, this activity decreased the sense of hopelessness and helplessness.

*Slowly, but steadily, I turned into a mindset like: ‘Well, it has to be like training for the Olympics’, I thought, ‘to get-, to come back. These Olympic-, the ones training for the Olympics, they don’t either feel like going out (.) exercising when it’s pouring down, and they don’t feel like eating all the time, or resting all the time, but’ – (2s) That’s how my thoughts were going, when I DIDN’T want to go for my walk, when I DIDN’T want to eat lunch, when I DIDN’T want to rest on the couch. (#7)*

Some quickly moved on to the next step, while others stayed on this quest for a long time before finding something that could work for them. The hope for recovery was very fragile, and it took a lot of effort and motivation to pursue the quest for recovery.

### The turning point: finding hope and a solid base to depart from

At some time in each story, there is a turning point, involving a new understanding of the illness, leading to less fear of debilitating symptoms, particularly fatigue. The new understanding is described in different ways, but generally entails a model of mind-body-interaction, and is accompanied by a new sense of self and agency. These new insights provided hope and motivation to search for and find new points of departure to explore possible ways to regain health. In some stories, the turning point was described as a sudden insight, like a “eureka moment”, whereas in others it was a more gradual turn.

In the stories, two types of events were mentioned that brought hope and constituted a turning point. First, hearing and reading about others who have recovered were described as very important, as they brought hope. Until then, almost everything that the interviewees had heard about their condition was that there was no cure or too little knowledge to understand what was going on. Interviewees expressed that the recovery stories shared by others with similar symptoms and limitations instilled a sense of hope and belief in the possibility of their own recovery.

*If you read in the right places on the internet, you will find many people who HAVE found ways out. And it is very hard to read several such stories and NOT feel that this must be possible for me too. Eeh (2s) And than you might have the little motivation needed. (#5)*

Second, experiences of being able to do something, as a result of a new understanding of the illness, also brought hope. This, in turn, seemed to have triggered curiosity and openness for a shift in the mindset of the interviewees and an emerging new relationship with their bodies.

*And it was there and then it HIT me that: Okay, my body maybe wants the best for me (1s) even with the illness. It actually might be trying to communicate something. Or protect me from whatever it might be. (#4)*

This new mindset is in some stories described as resonating well with interviewees’ prior knowledge, experience, and personality. In some cases it is described in terms of someone trustworthy from “outside” providing helpful suggestions. However, there are also descriptions of inner struggles before letting go of the earlier convictions.

*That really created this feeling of that something, something is wrong with me. Something is broken. Ehh (2s) so that narrative was deeply rooted in me. Eeh, and I think what happened was that, which took a very long time, was that it dissolved by the things I read and learnt. I understood that this- One can feel like this (1s) just because the nervous system CAN work like that. It’s not supposed to work like that, but it can get stuck in that state, kind of. (#5)*

### Searching, testing and evaluating

For the interviewees, the development of a new mindset implied that a new step towards more focused and comprehensive recovery work could be taken. The interviewees’ sense of being more in control of their illness and symptoms opened new possibilities for them to explore new strategies and behaviors for improved health. Most importantly, it seems that for the interviewees, acquiring/developing a new explanatory model for the symptoms provided a map to follow offering new possible pathways to improve health.

Anchored by a new understanding of the fatigue condition, this part of the recovery process was by all interviewees described as a process of testing and evaluating different new behaviors and strategies. The main questions the interviewees posed were “Does it work?”, “Does it improve my functioning?” Participants described themselves as rational and goal-oriented agents. They attempted online brain retraining programs, yoga, mindfulness exercises, vagus nerve stimulation, and visualization techniques. If a method worked, it strengthened their new understanding of the condition and being on the right track, so they continued trying out different methods. Even if the interviewees described setbacks with symptom flare ups, they were not perceived as frightening anymore.

*But (1s) it convinced me that when I felt symptoms (2s) ehh, they were real but not dangerous. It wasn’t that there was something wrong with me. […] When THAT really sank in, it became more like, if I experienced symptoms, if I went out and tested (.) a run, it became more of a PRACTICE. It wasn’t like—(1s) It wasn’t this DANGEROUS* thing *anymore. (3s) So (2s) I was able to (2s) somehow start calming myself down instead. (#5)*

In some of the interview stories, participants described a rapid return to health after discovering a helpful program to follow. However, more commonly, the recovery process involved a long period of a rather systematic searching, testing, and evaluating of various strategies and measures aimed at improving health and return to ordinary life.

*But I found these yeah, pretty big groups and communities on social media that actually supported each other so much. And helped each other, and people shared their experiences—like, ‘This worked for me, and this worked for me.’ Ehh, so I decided to kind of compile everything. Everyone I had PERSONALLY been in contact with, and everyone I had seen. And just, okay, what’s, what’s the common denominator in HOW they got sick and (1s) how they then recovered or got better? And so I did that. I ended up with like ten things and I thought, well, I’ll just do all of them. Ehh, and then I also noticed, very, very,* very *clearly, that everyone had the EXACT same way into this. And that they had the EXACT same way out of it. (#5)*

This systematic work was something the participants by and large did on their own, in most cases without support from health care, and sometimes with support from friends, family, and online communities. Some participants discovered that what they defined as unhealthy relationships stood in their way to recovery, and consequently they made decisions to try to change their social environments.

Even if the interviewees described hope and a conviction of being on the right track, progress was far from linear in their stories; many described how they experienced many discouraging setbacks along the way.

*And that’s something I’ve worked on—a sense of acceptance for the time it takes. Repeatedly sometimes every day, reminding myself that it* needs *to take time. It* will *take time. I wanted progress* now*, and as long as I didn’t accept that it takes time, at least for me, I ended up pushing a little too much. When I thought it should go a bit faster, I tried to increase the pace a bit more. But it always ended with me getting worse instead. So every time I lost that sense of acceptance, as I see it, I pushed myself and either got worse or my progress stalled. And then I had to go back and ask myself: What have I actually learned? Right, I need to do this and that, and it* will *take time. And when I regained that acceptance of time, that’s when I could start improving again. Then maybe a few weeks would pass, and I’d start pushing again. And then I had to slow down. (#13)*

Thus, recovery work is described as very demanding, and after some initial progress, many interviewees described how it was sometimes difficult to keep it up. The feeling of having the power to change one’s own health gave a responsibility, which was also experienced as a burden that got in the way of recovery by two interviewees.

### Regaining a life

Over time and gradually, the participants found ways to improve their physical, social, and psychological functions and well-being. Typically, this was a process that took many months, often years, although with some variation. The strong focus on recovery work faded away, and an orientation emerged towards living one’s life instead of being on a quest. Some have even described this in terms of a second turning point.

*Ehh, and it was really only when I TRULY accepted that this takes TIME that things actually started to happen. But as long as I was trying to speed it UP, as long as you RUSH, you’re in fight or flight. That activates the whole nervous system. And that keeps you in this heightened state. So* when *I* was *able to (2s) SETTLE and think, okay, what can I do here and now to feel good? How can I have a good DAY DESPITE or WITH post-COVID? Eh, that’s when I made sure that every DAY counted, instead of thinking I would just rehab for six months and THEN start living. Instead, I could shift my mindset to realizing that I’m living* now*. (#13)*

Leaving the state of being on a quest for recovery and testing and evaluating possibilities did not mean a return to life as it was before the illness. Almost all interviewees described the recovery process as personal growth. In retrospect, many participants described becoming aware of how earlier life experiences and behavioral patterns made them vulnerable to developing fatigue. It might be about being more in touch with one’s own feelings, stop being a “doer” in all situations, seek therapy to treat earlier psychological traumas, prioritize one’s own needs, or starting social media communities to help others. In some cases, new experiences lead to a change of workplace or career (e.g. becoming a health coach), where they tried to make active use of new insights.

## Discussion

The aim of the study was to deepen the understanding of the recovery process among individuals who had recovered from persistent fatigue. This was achieved by interviewing former patients about their recovery process and analyzing their narratives to develop a comprehensive model of recovery in persistent fatigue. From their stories, five key steps in the recovery process were identified: (1) the starting point and the health impasse; (2) quest; (3) turning point; (4) searching, testing, and evaluating; and (5) regaining a life. In this discussion, we address six issues pertaining to this study.

**Figure 1.**
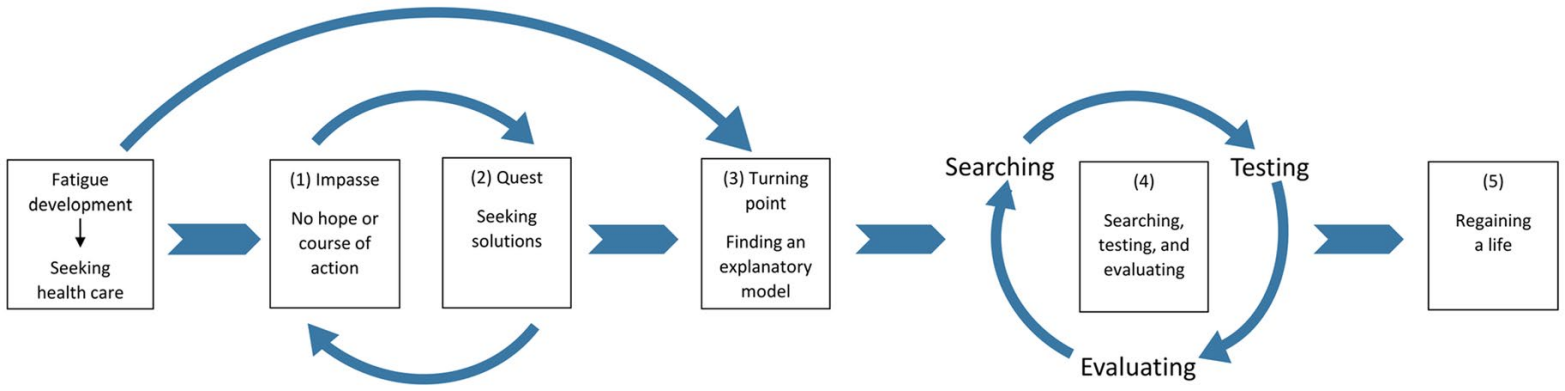
A model of the nonlinear recovery process as a sequence of events constituting a learning process.

First, the systematic analysis of all the interviewees’ stories revealed a distinct pattern: the recovery process as told by the interviewees consisted of **sequences of events** indicating the stepwise pattern. Each step consisted of progressing attempts to cope with the fatigue, eventually resulting in a sense of being recovered (cf. Figure 1). Even though each recovery narrative has unique features, it was striking how similar the stepwise pattern of this process was in the stories told by the interviewees, despite different diagnostic backgrounds. The duration and difficulty of each step varied, but the overall structure of this process seemed very similar between the narratives. All describe a turning point that involves finding hope and a new understanding of their fatigue condition, which offers new opportunities for action in the ensuing recovery journey. The findings from this study further indicate that the same step-wise pattern was found regardless of gender,

Second, the stepwise recovery process identified in this study could be described as a learning process in which the interviewees moved from experiencing helplessness towards a sense of agency (see Figure 1). As in Brown et al.’s study [35] and Bakken et al.’s [33] the recovery process can be described as self-driven. Consistent with our findings, Bakken et al. emphasize the importance of the patient developing a new, helpful understanding of symptoms and the body, which in turn supports the development of a new sense of agency. Krabbe et al. [34] described this as a gradual process originating from exploring bodily limits and capacities rather than a change in mindset as a distinct turning point. In contrast, our study suggests that finding an explanatory model for debilitating symptoms served as a turning point, providing the interviewees with a map for the rest of their recovery journey, despite facing many challenges (Figure 1). Especially for those diagnosed with CFS/ME and PCC, there was a shift in the conceptualization of their condition towards an illness model integrating processes of mind and body.

The later stage of the learning process is organized around a circle of searching, testing and evaluating where hope is a central component (Cf [25]). This stage of the process resembles that of learning health systems, where data are systematically gathered within health care to create evidence and then the most promising evidence is applied to the system [47]. Similar to a learning health system, the interviewees tested and evaluated strategies and implemented the interventions they found helpful to improve their health.

Third, what stands out in the stories about the recovery process is that the interviewees repeatedly expressed how they to a large extent had to do recovery learning work **on their own**, without support from health care and mainly more as consumers of social media posts than as active members of online communities. It is predominantly a recovery process described as taking place outside or even despite the health care system, which raises issues about the social and structural aspects of the recovery process. Two participants diagnosed with ED were a notable exception since they had received a stress-physiological explanatory model for the symptoms at the same time as their diagnosis, which seems to have facilitated their recovery process. In addition, they were offered a helpful rehabilitation program. The support from health care meant that they skipped the steps of “No hope” and “Quest” and moved directly to the turning point. However, a different picture is presented in a recent study of by Ellbin et al. [48]. They found that ED patients experienced dismissive encounters with health care, creating an experience that could negatively influence the recovery process.

Fourth, the absence of scientific consensus regarding etiology and the lack of access to evidence-based interventions contribute to confusion and a lack of hope both in patients with fatigue and health care professionals. Even though the evidence base for effective treatments for fatigue and other persistent symptoms is growing [49–51], public discourse is still dominated by narratives about persistent fatigue as an untreatable condition with a poor prognosis. Such perceptions are still widespread within health care and could help explain why few interviewees reported finding a way to recovery within the health care system.

Fifth, given that the majority of the interviewees did not experience any support from health care services, there is a risk that the recovery process is perceived as an entirely individual endeavor. It is important to acknowledge that not all individuals have access to the resources required to make necessary changes in their lives or to invent their own learning health system. Vulnerable groups may have greater difficulties navigating the health care system, thereby being disproportionately hindered from pursuing the road to recovery. As Karadzhov [52] argues, overlooking or downplaying the socio-structural conditions of individuals’ lives may reinforce a reductionist view of recovery – one that frames it as an atomized, individualized process. In light of the absence of clinical guidelines and recommended treatments, it is essential to expand our understanding of how patients with persistent fatigue seek and experience health care interactions, as well as alternative forms of treatment and support. A recent study found that patients diagnosed with CFS/ME invested considerable time and energy in identifying, implementing and adapting therapeutic interventions – often without the guidance of medical professionals [31].

Finally, there are many similarities and differences in relation to research on mental health recovery processes. Similar to mental health recovery, fatigue recovery is a transformative process of the self. As described by Dell et al. [53], it is about moving from a state of powerlessness and hopelessness to agency and being in charge of managing one’s health challenges. The different patient groups also share experiences of lack of access to effective treatment and experiencing treatment settings as unsupportive. One important difference is how severe fatigue seems to prevent interviewees from developing meaningful roles and activities and accessing social support with peers and professionals, which has been shown to be central in mental health recovery processes [53]. In our study, it was striking how much the recovery process was described as taking place without support from others, although social media platforms could provide some sense of connectedness.

### Clinical implications

This study on the experience of recovering from persistent fatigue has important clinical implications. The stepwise recovery process, as described in the analysis and summarized in Figure 1, highlights the importance of a shift in symptom perception and understanding to identify new action possibilities for patients, which may ultimately lead to recovery. This finding is in line with the transdiagnostic approach to understanding and treating persistent physical symptoms, including persistent fatigue, which was recently presented by Löwe et al. [54]. As a central part of basic care, Löwe et al. stress the importance of person-centered communication techniques that validate and explain symptoms to the patient in a way that offers possibilities to change the process and avoid the induction of dysfunctional expectations about symptom development and treatment effects. When basic care is insufficient, person-centered treatments can be added. In short, the authors suggest that health services must provide understanding, hope, and a map to show the way forward. When that is insufficient, a personal guide is needed to support the patient on the journey to recovery.

An approach like the one described above could likely facilitate the recovery process for individuals experiencing persistent fatigue, so that patients would be spared hopelessness and time lost in a loop of ‘no hope’ and ‘quest’. However, conflicting understandings of persistent fatigue in research and clinical practice make the implementation of the treatment principles of Löwe et al. more difficult than it might otherwise be. Currently, the contested nature of conditions such as CFS/ME, ED and PCC likely contributes to individuals becoming stuck in fear and uncertainty regarding their prognosis.

### Methodological considerations

It is difficult to assess how representative the interviewed sample is for people who have recovered, since there is little research on this topic. Although there was an explicit aim to try to recruit participants from different social and educational backgrounds, in the end, the majority had university degrees and had been working full-time before developing fatigue. Given the resources needed to go through the recovery process, predominantly outside of the health care system, this is hardly surprising. They all have the intellectual ability to find information and apply it to their own situations. They often also had economic means to pay for brain retraining programs outside of health care. It seems likely that the high levels of education, health literacy and digital competence among the participants probably has less to do with a sample bias and more to do with who has the resources needed to recover outside of the health care system. Most reported being healthy before developing persistent fatigue, which from our clinical experience indicates belonging to a subgroup of patients with a somewhat lower degree of complexity, compared with patients with a history of several comorbidities, as well as distressing economic and social situations.

Some interviews were, due to practical reasons, conducted digitally. This might have influenced the depth or emotional expressiveness of the interviews. However, we could not see that the digital interviews differed from the face-to-face interviews in this respect. It appears that after the pandemic of Covid-19, meeting new people online has become a normal form of social interaction, that both the interviewer and those interviewed felt comfortable with, even when talking about highly emotional issues.

### Future directions

Further research is needed within the field of persistent fatigue and other persistent physical symptoms [54]. We would like to highlight the need to study the implementation of treatment and communication strategies for persistent physical symptoms in clinical practice. Health care providers need helpful explanatory models, based on the latest scientific evidence, to raise hope and provide the map patients need to start their recovery journey. One way to increase knowledge of how this could be done is by further studying the experiences of those who have recovered. There is also a need to study the experiences of those who, despite finding helpful explanatory models and putting much effort into their own recovery, do not reach the point at which they define themselves as recovered.

## Conclusion

The results of this study highlight how participants’ narratives provide insight into their recovery process and how these contribute to a new understanding within the field of persistent fatigue.

The recovery narratives were strikingly similar, often highlighting a turning point when participants received an explanatory model for their symptoms. This model provided hope, a sense of agency, and reduced fear by signaling safety. Recovery stories from others also played a crucial role in fostering belief in the possibility of personal recovery. The journey toward improvement frequently occurred outside of formal health care settings, with or without support from others. The participants in this study demonstrated notable resourcefulness, which may set them apart from those who do not recover from persistent fatigue, having navigated their path largely on their own, without structured support from health care services.

## Data Availability

The interview data generated and analyzed during this study contain confidential and potentially identifiable information. Owing to ethical and privacy concerns, these data cannot be made publicly available. However, de-identified excerpts or further details may be provided by the corresponding author upon reasonable request and upon institutional approval.
